# The regulatory role of eosinophils in adipose tissue depends on autophagy

**DOI:** 10.3389/fimmu.2023.1331151

**Published:** 2024-01-03

**Authors:** Aref Hosseini, Nina Germic, Nikita Markov, Darko Stojkov, Kevin Oberson, Shida Yousefi, Hans-Uwe Simon

**Affiliations:** ^1^ Institute of Pharmacology, University of Bern, Bern, Switzerland; ^2^ Institute of Biochemistry, Brandenburg Medical School, Neuruppin, Germany

**Keywords:** adipose tissue, arginase 2, *Atg5*, autophagy, beiging, eosinophils, macrophages

## Abstract

**Introduction:**

Obesity is a metabolic condition that elevates the risk of all-cause mortality. Brown and beige adipose tissues, known for their thermogenic properties, offer potential therapeutic targets for combating obesity. Recent reports highlight the role of immune cells, including eosinophils, in adipose tissue homeostasis, while the underlying mechanisms are poorly understood.

**Methods:**

To study the role of autophagy in eosinophils in this process, we used a genetic mouse model lacking autophagy-associated protein 5 (*Atg5*), specifically within the eosinophil lineage (*Atg5* ^eoΔ^).

**Results:**

The absence of *Atg5* in eosinophils led to increased body weight, impaired glucose metabolism, and alterations in the cellular architecture of adipose tissue. Our findings indicate that *Atg5* modulates the functional activity of eosinophils within adipose tissue rather than their abundance. Moreover, RNA-seq analysis revealed upregulation of arginase 2 *(Arg2)* in *Atg5*-knockout eosinophils. Increased *Arg2* activity was shown to suppress adipocyte beiging. Furthermore, we observed enrichment of the purine pathway in the absence of *Atg5* in eosinophils, leading to a pro-inflammatory shift in macrophages and a further reduction in beiging.

**Discussion:**

The data shed light on the importance of autophagy in eosinophils and its impact on adipose tissue homeostasis by suppressing *Arg2* expression and limiting inflammation in adipose tissue.

## 1 Introduction

Eosinophils are granulocytes characterized by their strong affinity for the acidic dye eosin (1), which originate from the bone marrow and possess versatile effector functions within both innate and adaptive immunity (2). Eosinophils migrate from the bloodstream into different tissues during physiological and pathological states. They carry out their diverse functions by synthesizing and releasing various granule proteins and pro-inflammatory mediators. Despite their presence in all vertebrate species, the specific functional role of eosinophils continues to be a matter of ongoing debate (3). However, the persistence of the eosinophil lineage across all vertebrate species throughout evolution points to a crucial role of eosinophils in health and disease (2). For instance, eosinophils are believed to play a role in host defense against various pathogens. Moreover, they have been reported to be involved in tissue homeostasis, including within adipose tissue (AT) (4).

Autophagy is a strictly regulated catabolic process wherein cellular contents are engulfed within double-membrane vesicles known as autophagosomes that fuse to lysosomes for degradation by proteolytic lysosomal enzymes (5). Autophagy-associated protein 5 (ATG5) plays a crucial role as an essential component within the autophagic pathway (6). Interestingly, autophagy is involved in the regulation of both innate and adaptive immune responses. For example, ATG5 and autophagy have been shown to regulate the process of differentiation of various cell types, including innate lymphoid cells (7), neutrophils (8), B cells (9), and plasma cells (10). Moreover, ATG5 plays a pivotal role in a wide array of processes, encompassing clearance of pathogens (11, 12), cytokine secretion (13), and facilitation of antigen presentation (14, 15). Furthermore, we have previously demonstrated a role for ATG5 and autophagy in both eosinopoiesis and eosinophil effector functions (16).

Three discrete AT types exhibit differences in function, structure, and anatomical distribution: white adipose tissue (WAT), brown adipose tissue (BAT), and beige adipose tissue (BeAT). The WAT stores energy in the form of triglycerides within adipocytes, predominantly located in depots such as the inguinal and visceral regions. Conversely, BAT plays a crucial role in heat generation and is particularly abundant in hibernating animals, rodents, and human infants. Brown adipocytes have abundant mitochondria expressing uncoupling protein 1 (UCP1), enabling uncoupled respiration, which releases stored energy as heat instead of ATP conversion (17). On the other hand, BeAT is located within WAT depots while exhibiting thermogenic characteristics similar to BAT (18). The activity of BAT and BeAT is linked to improved glucose and lipid metabolism and reduced adiposity in both mice and humans. This association suggests a promising therapeutic approach for addressing obesity and related metabolic disorders (19).

Several recent reports suggest a role of AT resident immune cells, notably those associated with type 2 immunity, for BeAT stimulation and energy dissipation. While eosinophils have been involved in this process (20, 21), the precise biological mechanisms governing their involvement remain partially understood. Obese mice possess a reduced number of AT-eosinophils compared to their lean counterparts. A transgenic murine model with a deficiency in eosinophils, denoted by ΔdblGata, exhibits excessive weight gain when subjected to a high-calorie diet (21). This model suggests that AT-eosinophils may play a role in facilitating adipocyte maturation, potentially alleviating diabetic complications associated with diet-induced obesity (22). In contrast, IL-5 overexpressing mice show elevated eosinophil levels and resistance to diet-induced obesity (21). Moreover, a recent investigation revealed that augmenting AT-eosinophils through the administration of recombinant IL-5 failed to yield the expected metabolic advantages, suggesting that eosinophil function may extend beyond their mere abundance (23).

In this study, we demonstrate the critical role of *Atg5* and autophagy in eosinophils within the context of adipose tissue using *Atg5* ^eoΔ^ mice. Interestingly, despite observing elevated eosinophil levels in adipose tissue in this genetic model, we observed increased body weight and impaired glucose metabolism. Our findings highlight the pivotal role of autophagy in eosinophils in preserving adipose tissue homeostasis and promoting beiging through the inhibition of *Arg2* expression and the reduction of inflammation.

## 2 Materials and methods

### 2.1 Animal

Animal experiments were carried out following approval by the Veterinary Office of the Canton of Bern and conducted under Swiss federal legislation on animal welfare under animal license number BE26/2021. The generation of *Atg5* ^flox/flox^eo*Cre* mice, referred to as *Atg5* ^eoΔ^, was achieved by crossing *Atg5* ^flox/flox^ mice (24) (*Atg5* ^tm1Myok^) with eo*Cre* mice (25) (*Epx* ^tm1.1(cre)Jlee^), as previously published (26). The *Atg5* ^flox/flox^ mice was provided by Dr. Christian Münz, University of Zürich, Switzerland. *Atg5* ^flox/flox^eo*CreIl5* ^tg^ mice, denoted as *Atg5* ^eoΔ^ *Il5* ^tg^, were produced through the crossbreeding of *Atg5* ^eoΔ^ mice with *Il5* ^tg^ [Tg(*Cd3d-Il5)NJ.1638Nal*] mice (27) to augment the eosinophil number in order to obtain sufficient number of eosinophils for *in vitro* assays. The eo*Cre* and *Il5* ^tg^ mice were kindly provided by Dr. Jamie J. Lee, the Mayo Clinic, Phoenix, USA. Throughout the study the Eo*Cre* mice is designated as the control group, and eo*CreIl5* ^tg^ mice as Ctrl*Il5* ^tg^.

### 2.2 HFD-induced obesity and metabolic analyses

Six-week-old female mice were fed a high-fat diet (HFD) (60% kcal from fat), (Cat # E15742-34 Ssniff Spezialdiäten GmbH, Soest, Germany) or a normal diet, (Cat # N3436, Kliba NAFAG, Kaiseraugst, Switzerland) for ten weeks, during which body weight was consistently measured on a weekly basis. Food consumption was assessed within metabolic cages. For the intraperitoneal glucose tolerance test (IP GTT), animals underwent a morning fast lasting six hours, from 8:00 to 14:00. Blood samples were collected from the tail vein, and the animals received an intraperitoneal injection of 1.5 g glucose (D-(+)-Glucose Solution, 45%), (Cat # G8769, Sigma-Aldrich, Buchs, Switzerland) per kg body weight. Blood samples were subsequently drawn at 0, 15, 30, 45, 60, 90, 105, and 120 minutes. Blood glucose levels were assessed using a glucometer (Accu-Chek), (Roche Diagnostics, Rotkreuz, Switzerland). For the insulin tolerance test (ITT), following a six-hour morning fast, the intraperitoneal injection of 0.75 IU insulin (Actrapid), (Novo Nordisk, Bagsvaerd, Denmark) was administered. Blood glucose levels were monitored at identical time intervals, consistent with those employed in the glucose tolerance test (GTT), utilizing the same measurement method to comprehensively assess insulin sensitivity.

### 2.3 Glucose uptake assay

Six-week-old female mice underwent a 5-hour fasting period, followed by the intraperitoneal administration of 0.75 IU insulin (Actrapid, Novo Nordisk, Bagsvaerd, Denmark) per kg of body weight. After 10 minutes, 2-deoxyglucose (2-DG) (Cat # D8375, Sigma-Aldrich, Buchs, Switzerland) was intraperitoneally administered at a dose of 32.8 μg/g body weight, and inguinal AT, visceral AT, brown AT, liver, and muscle were collected 20 minutes later and washed with phosphate buffered saline (PBS). The 2-deoxyglucose-6-phosphate (2-DGP) levels were quantified using Glucose Uptake-Glo Assay Kit (Cat # J1341, Promega AG, Dübendorf, Switzerland), following the manufacturer’s recommended protocol. Specifically, 40 μg of tissue samples were lysed in 300 μL of stop buffer, homogenized using an Omni Tissue Homogenizer (Omni International, Kennesaw, USA) for 30 seconds, and subsequently mixed with 300 μL of neutralization buffer via vortexing. Afterward, 100 μL of the homogenized extract solution was transferred to a white, opaque 96-well plate, containing 100 μL of 2-DG6P detection reagent. After a 10-minute incubation period at room temperature in darkness, luminescence activity was recorded using a GloMax Explorer machine (Promega AG, Dübendorf, Switzerland). Pierce BCA protein assay kit (Cat # 23227, Thermo Fisher Scientific, distributed by LuBioScience GmbH, Lucerne, Switzerland) assay was employed to measure protein concentration of each sample to normalize the tissue input.

### 2.4 Histology and immunofluorescence staining

Inguinal AT samples underwent a series of preparation steps. Initially, the tissue was perfused with PBS, followed by fixation in 4% formalin for 24 hours and embedding in paraffin blocks. Subsequently, thin tissue sections, approximately 4-5 µm in thickness, were prepared from the formalin-fixed paraffin-embedded (FFPE) tissue blocks. Samples were deparaffinized using NeoClear (Cat # 1.09843, Merck Millipore, Darmstadt, Germany) and rehydrated through a series of graded ethanol dilutions ranging from 100% to 40%. For a broad assessment of tissue histology, the sections from control and *Atg5* ^eoΔ^ mice were subjected to staining with hematoxylin and eosin (H&E) solution. For immunofluorescence (IF) staining of inguinal AT from Ctrl*Il5* ^tg^ and *Atg5* ^eoΔ^ *Il5* ^tg^, the tissue sections were deparaffinized and rehydrated and subsequently the antigen retrieval was accomplished by boiling the samples in a 10 mM citrate buffer (pH 6.0) for 12 minutes. To reduce nonspecific binding, a blocking buffer composed of 1.9% bovine serum albumin (Sigma-Aldrich, Buchs, Switzerland), 25% human IgG polyvalent, 25% normal goat sera, and 1% ChromPure human IgG (Cat # 009-000-003, Jackson ImmunoResearch Laboratories Inc., Philadelphia, USA) in PBS, was applied and allowed to incubate for one hour at room temperature. The staining process involved incubating the tissue sections with a monoclonal mouse anti-EPX antibody (1:500; clone MM25-82.2, Lee Laboratories, Mayo Clinic, USA), and subsequently, a secondary antibody, Alexa-488 goat-anti mouse IgG (Cat # A11017, Thermo Fisher Scientific, distributed by LuBioScience GmbH, Lucerne, Switzerland) (5 μg/ml), was applied for one hour at room temperature. Nuclei were stained with propidium iodide (PI) solution (1 μg/mL) for an additional 10 minutes, and the tissue samples were mounted using ProLong Gold Antifade mounting medium (Cat # P10144, Thermo Fisher Scientific). Lastly, the slides were examined, and images were acquired using both the confocal laser scanning microscope LSM 800 (Carl Zeiss Micro Imaging, Jena, Germany) and the automatic digital slide scanner Panoramic MIDI II (3DHISTECH, Budapest, Hungary). To perform a quantitative analysis, EPX^+^ infiltrating cells were manually counted in 10 randomly selected high-power fields (HPF), each covering an area of 22.5×10^-3^ mm². The purity and morphology of the isolated mouse eosinophils were evaluated using Hemacolor Rapid (Cat# 111661, Sigma), followed by light microscopy analysis using a 60X objective on a BZ-X810 microscope (Keyence, Osaka, Japan). The purity of isolated blood eosinophils exceeded 95%.

### 2.5 Quantitative PCR

RNA from the samples was extracted using the RNeasy Plus Mini Kit (Cat # 74134, Qiagen, Hilden Germany), following the manufacturer’s instructions. Subsequently, cDNA was synthesized using 1000 ng of RNA, utilizing the iScript™ gDNA Clear cDNA Synthesis Kit (Cat # 1725035, Bio-Rad, Hercules, USA). Transcription levels were assessed in duplicate using iTaq™ Universal SYBR^®^ Green Supermix (Cat # 1725121, Bio-Rad) and specific primer pairs (**Supplementary Table 1**) in the T100 Gradient Thermal Cycler (Bio-Rad). The primers were synthesized by Microsynth AG (Balgach, Switzerland) and were utilized at a final concentration of 250 nM. The amplification protocol involved an initial activation step at 95°C for 3 minutes, followed by 40 cycles of denaturation at 95°C for 10 seconds, annealing at 58°C for 30 seconds, and extension at 72°C for 10 seconds employing the CFX Connect Real-Time PCR Detection System (Bio-Rad).

### 2.6 Isolation of mouse bone marrow and blood eosinophils

For *in vitro* experiments, eosinophils were extracted from the bone marrow and peripheral blood of Ctrl*Il5* ^tg^ and *Atg5* ^eoΔ^ *Il5* ^tg^ mice as previously described (16). In brief, bone marrow cells were obtained by flushing the femurs and tibias with a medium consisting of 2% FCS in PBS, using a 26-gauge needle, and then filtering the cells through a sterile 70 µm nylon cell strainer, as previously described (28). The bone marrow cell count was determined, and they were resuspended at a concentration of 1×10^8^ cells/ml in medium (2% FCS in PBS) for eosinophil isolation. T cells, B cells, macrophages, and neutrophils were depleted using specific antibodies. The monoclonal rat anti-CD8α-PE (clone 53-6.7, Cat # 130- 102-595); anti-CD19-PE (clone 6D5, Cat # 130-102-598); anti-CD90.2-PE (clone 30-H12, Cat # 130-102-489); and anti-Ly6G-PE (clone 1A8, Cat # 130-102-392) were purchased from Miltenyi Biotec (Bergisch Gladbach, Germany) (each at 0.3 µg/ml concentration). The isolation of the cells was performed using the EasySep™ Mouse PE Positive Selection Kit II (Cat # 17666, Stemcell Technologies, Vancouver, Canada) following the manufacturer’s instructions. The purity of mouse eosinophils was assessed to be 90-95% using cytospin and staining with the Hemacolor Rapid staining kit (Cat # 111661, Merck Millipore, Darmstadt, Germany), followed by light microscopic analysis. The eosinophils isolation from peripheral blood was performed as previously reported (29). Briefly, subsequent to dilution of the blood with PBS at a 1:1 ratio, it was gently layered over Histopaque 1119 (density of 1.119 g/mL) (Sigma-Aldrich, Buchs, Switzerland) and centrifuged at 800g for 20 minutes, resulting in an augmented eosinophil layer. The eosinophil-containing interface was briefly treated with ice-cold distilled water to lyse erythrocytes and then washed with PBS before isolation. Following cell counting and resuspension at a concentration of 1×10^8^ cells/ml, eosinophil purification was conducted as described above. To study the impact of increased arginase activity of *Atg5*-knockout eosinophils on mature adipocytes, we cultured control or *Atg5*-knockout eosinophils in L-arginine free DMEM medium (Cat # CC927-0500, GeneDireX, Keelung, Taiwan) supplemented with 200 μmol L-arginine (Cat # A6969, Sigma-Aldrich) in the presence or absence of the arginase inhibitor, 1 mM ABH hydrochloride (6-Borono-L-norleucine hydrochloride), (Cat # sml1466, Sigma-Aldrich). After 5 hours of incubation, the supernatants were collected and used to treat the adipocytes for an additional five hours of incubation. Finally, adipocytes were collected to analyze the expression level of thermogenic genes.

### 2.7 RNA-seq method

The quantity and quality of the purified total RNA was assessed using a Thermo Fisher Scientific Qubit 3.0 fluorometer with the Qubit RNA BR Assay Kit (Cat # Q10211, Thermo Fisher Scientific) and an Advanced Analytical Fragment Analyzer System using a Fragment Analyzer RNA Kit (Cat # DNF-471, Agilent Technologies, Santa Clara, USA), respectively. Sequencing libraries were constructed using an Illumina TruSeq Stranded mRNA Library Prep kit (20020595, Illumina, San Diego, USA) in combination with TruSeq RNA UD Indexes (Cat # 20022371, Illumina) according to Illumina’s guidelines. The cDNA libraries were evaluated using a Thermo Fisher Scientific Qubit 3.0 fluorometer with the Qubit dsDNA HS Assay Kit (Cat # Q32854, Thermo Fisher Scientific) and an Agilent Fragment Analyzer (Agilent) with an HS NGS Fragment Kit (Cat # DNF-474, Agilent), respectively. Pooled cDNA libraries were sequenced 100 bp single-end on one lane of an Illumina HiSeq 3000 instrument (Illumina). All base call files were demultiplexed and converted into FASTQ files using Illumina bcl2fastq conversion software. The quality control assessments, generation of libraries, and sequencing were conducted by the Next Generation Sequencing Platform, University of Bern.

### 2.8 RNA-seq analysis

The RNA-seq data quality was evaluated using Fastqc v. 0.11.9. Afterward, the reads were aligned to the reference genome using HISAT2 v. 2.2.1 (30). To determine the number of reads associated with each gene based on the genome annotation (Mus_musculus.GRCm39.104), FeatureCounts v. 2.0.1 (31) was utilized. Subsequent data analysis was conducted in R v. 4.1.0. Differential expression analysis was carried out using the Bioconductor package DESeq2 v. 1.32.0 (32) with default settings. For visualization, the results were presented using EnhancedVolcano v. 1.10.0 (33) and pheatmap v. 1.0.12 (34). Gene set enrichment analysis (GSEA) was also performed for further analyses (35).

### 2.9 Immunoblotting

Immunoblotting was performed as previously described (16). Briefly, Cell lysates were prepared by resuspending the cell pellet in lysis buffer. Shortly before use, a protease inhibitor cocktail (Cat # P8340, Sigma-Aldrich, Buchs, Switzerland) and 1 mM PMSF were added to the lysis buffer. The protein concentration was measured using a Pierce BCA protein assay kit (Cat # 23227, Thermo Fisher Scientific). Extracted proteins (50 μg) were denatured and separated on 12% SERVAGel TG PRiME gel (Cat # 43266, SERVA Electrophoresis, Heidelberg, Germany), followed by protein transfer onto Immobilon-P PVDF membrane (Cat # ipvh304f0, Merck Millipore, Darmstadt, Germany). Membranes were blocked in 5% non-fat milk in TBST (0.1% Tween 20 in 20 mM Tris and 150 mM NaCl [pH 7.6]) for 1 hour and incubated with primary antibodies at 4°C overnight. The antibodies used for immunoblotting were monoclonal mouse anti-ATG5 (clone 7C6, Cat # 0262-100; Nanotools, Teningen, Germany), polyclonal rabbit anti-UCP1 (ab10983, Abcam, Cambridge, USA), monoclonal rabbit anti-ARG2 (Cat # 55003, Cell Signaling Technology, Danvers, USA), polyclonal rabbit anti-β-actin (Cat # AAN01-A; Cytoskeleton, Denver, USA). The membranes were washed with TBST three times and incubated with the corresponding HRP-conjugated secondary antibody. Horseradish peroxidase (HRP)-conjugated sheep anti-mouse (Cat # NA931V, GE Healthcare Life Sciences, Little Chalfont, UK) and HRP-conjugated donkey anti-rabbit (Cat # NA934V, GE Healthcare Life Sciences, Little Chalfont, UK) antibodies. The signal was detected using the Immobilon Forte Western HRP substrate (Cat # wbluf0500, Merck Millipore, Burlington, USA) and images acquired on the Odyssey Fc Imaging System (LI-COR Biosciences, Lincoln, USA).

### 2.10 Flow cytometry

Stromal vascular fraction (SVF) cells were harvested from the inguinal AT, and to block Fc receptors, they were incubated with FC block (Cat # 156604, TruStain FcX PLUS, anti-mouse CD16/32, BioLegend, London, UK) for 10 minutes. For the macrophages panel, cells were stained with anti-CD86-APC-R700 (Cat # 565479, BD Biosciences, San Jose, USA), anti-F4/80-APC antibody (Cat # 123116, BioLegend), and anti-CD11b PE-Cy7 (Cat # 25-0112-81, eBioscience, San Diego, USA). For the eosinophils panels, anti-Siglec-F BV421 (Cat # 562681, BD Biosciences), anti-CD11b APC (Cat # 101212, BioLegend), anti-CD63 PE(Cat# 564222, BD Biosciences), and anti-CD193 (CCR3) Alexa 647 (Cat# 144508, BioLegend) were used. After staining, cells were washed with a washing buffer (2% FCS in PBS) and analyzed using a BD FACSLyric™ flow cytometer (BD Biosciences).

### 2.11 Macrophage differentiation and polarization

Mouse bone marrow cells were harvested from the femurs and tibias. To initiate differentiation, 5.5 × 10^6^ bone marrow cells were cultured in a T75 flask in 12 ml of complete Iscove’s Modified Dulbecco’s Media (IMDM) (Cat # I3390-500ML, Sigma-Aldrich, Buchs, Switzerland) containing 2 mM L-glutamine, 100 U/ml penicillin/streptomycin, 20 mM 2-mercaptoethanol, and 10% FCS and supplemented with 30% L929 cell supernatant for eight days. An additional 8 ml of the same media without 2-mercaptoethanol was added on day four of the culture. On the eighth day, the bone marrow-derived macrophages were washed, trypsinized, counted using a hemocytometer, and seeded. Subsequently, on the ninth day, the macrophages were subjected to stimulation for 24 hours with 100 ng/ml LPS (Cat # L6529, Sigma-Aldrich) and 20 ng/ml IL-4 (Cat # 214-14, PeproTech, London, UK) to induce M1 (pro-inflammatory) and M2 (anti-inflammatory) macrophages, respectively, and these were compared with unstimulated cells as M0. To assess the impact of eosinophils on macrophages, we polarized macrophages in the presence of control eosinophils, *Atg5*-knockout eosinophils, or media alone for 24 hours. Additionally, to neutralize IFN-γ, we introduced IFN-γ neutralization antibody (NAb) (clone XMG1.2, Cat # 505834, Biolegend, London, UK) into the system.

### 2.12 Isolation and differentiation of primary preadipocytes

The cultivation and differentiation of primary mouse preadipocytes were carried out following previously reported procedures (36). In brief, inguinal AT was dissected, finely chopped, and subjected to enzymatic digestion using a mixture of collagenase D (Cat # 11088866001, 1.5 U/mL, Roche, Mannheim, Germany) and dispase II (Cat # D4693, 2.4 U/mL, Sigma-Aldrich, Buchs, Switzerland), supplemented with 10 mM CaCl_2_, for approximately 15 to 20 minutes at 37°C with constant agitation. After digestion, the tissue suspension was filtered through a 100 μm cell strainer and then centrifuged at 400g for 5 minutes to pellet the cells. This cell pellet was subsequently resuspended and filtered through a 70 μm cell strainer, followed by another round of centrifugation to obtain the SVF. The SVF was collected for flow cytometry analyses when needed. The cell pellet was resuspended in a culture medium (consisting of DMEM/F12 GlutaMAX supplemented with 20% FBS and 100 U/ml penicillin/streptomycin) and plated in 6-well plates. To induce adipocyte differentiation, confluent cell cultures were exposed to an induction medium composed of DMEM/F-12 GlutaMAX supplemented with 10% FBS, 100 U/ml penicillin/streptomycin, 0.5 μg/mL insulin, 5 μM dexamethasone, 1 μM rosiglitazone, and 0.5 mM IBMX. Two days after induction, cells were transitioned to a maintenance medium, DMEM/F12 GlutaMAX supplemented with 10% FBS, 100 U/ml penicillin-streptomycin, and 0.5 μg/mL insulin for an additional four days, which was changed every other day during the subsequent four-day period. By the sixth day, the cells were prepared for experimental purposes. To assess the effect of different concentrations of L-arginine, mature adipocytes incubated in L-arginine free DMEM medium (Cat # CC927-0500, GeneDireX, Keelung, Taiwan) supplemented with various concentrations of L-arginine (Cat # A6969, Sigma-Aldrich) for five hours. Also, to evaluate the effect of the conditioned media of eosinophils alone or eosinophils-macrophages co-culture, adipocytes were treated with the supernatant of eosinophils or eosinophils-macrophages media for five hours.

### 2.13 ELISA measurements

The levels of IL-1β (Cat # 432604, Mouse IL-1β ELISA MAX™ Deluxe Set; BioLegend, London, UK) and IFNγ (Cat # 430804, Mouse IFN-γ ELISA MAX™ Deluxe Set; BioLegend) in the supernatant of the eosinophils-M1 macrophages were assessed using enzyme-linked immunosorbent assay (ELISA) as per the guidelines provided by the manufacturer.

### 2.14 Measurements of uric acid, ATP, and arginase activity

The uric acid concentration was determined using an enzymatic colorimetric method with a standard kit (Cat # MAK077, Sigma-Aldrich, Buchs, Switzerland) following the manufacturer’s instructions. To measure extracellular ATP release, the CellTiter-Glo^®^ luminescent assay (Cat # G7571, Promega AG, Dübendorf, Switzerland) was employed. Arginase activity was assessed by utilizing the Arginase Activity Assay Kit (Cat # MAK112, Sigma-Aldrich) in accordance with the manufacturer’s protocols.

### 2.15 Statistical analysis

Statistical analysis for all data was conducted using GraphPad Prism software (GraphPad Software Inc., San Diego, USA). The figures display mean values ± SEM, and the figure legends contain information about the number of independent experiments conducted (indicated by n). Statistical comparisons were carried out using the unpaired Student’s *t*-test, multiple *t*-test, and two-way ANOVA. Statistically significant results were defined as those with *p* values <0.05.

## 3 Results

### 3.1 Decreased beiging and increased adiposity in *Atg5* ^eoΔ^ mice

As described previously by Germic et al. (16), we crossed *Atg5* ^flox/flox^ mice with a knock-in strain of mice expressing *Cre* recombinase under the control of the eosinophil peroxidase (EPX) promoter, which is exclusively active in eosinophils (eo*Cre* mice). Heterozygous *eoCre* mice (*eoCre* ^+/-^) were used in all experiments as they exhibited adequate Cre recombinase expression for targeted gene excision while retaining half of the total EPX protein levels (25). The resulting conditional knockout mouse line in which *Atg5* is specifically deleted in the eosinophil lineage was designated *Atg5* ^eoΔ^. As eosinophils have been suggested to play a role in beiging (37), we used this mouse model to study the role of autophagy in eosinophils in this process.

**Figure 1:**
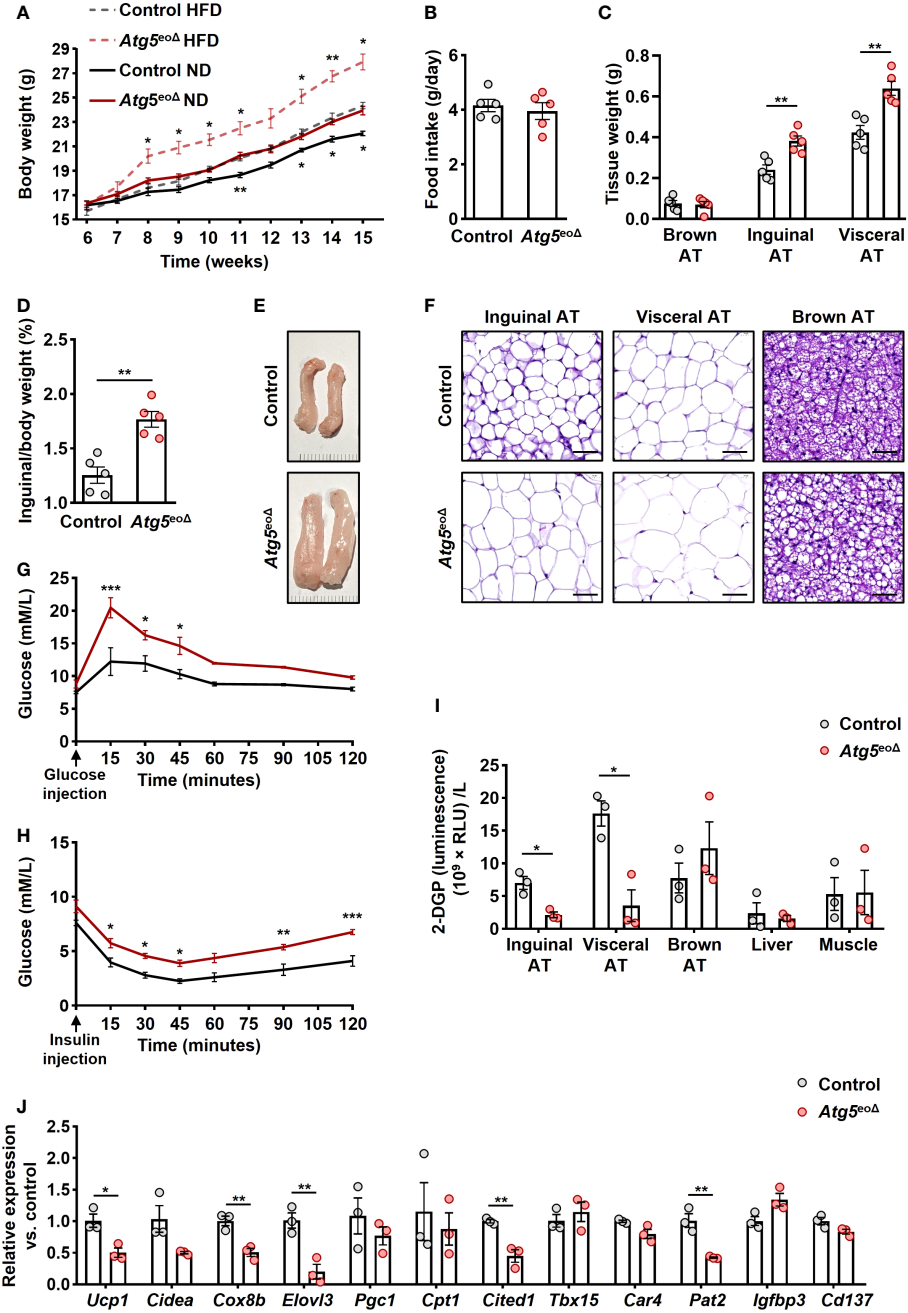
The impact of ATG5 deficiency in eosinophils on adiposity, adipose tissue morphology, and glucose metabolism. **(A)** Body weight. The body weight of female mice was recorded for ten weeks during the HFD and normal diet (n = 6-7). **(B)** Food intake. We measured the weight of food intake over a 24-hour period (n = 5). **(C)** ATs weight. The weight of inguinal, visceral, and brown AT of control and *Atg5* ^eoΔ^ male mice was measured at the age of 5 months (n = 5). **(D)** AT/body weight ratio. The ratio of inguinal AT to the total body weight of male mice examined at 5 months aged (n = 5). **(E)** Macroscopic images. Representative macroscopic images of inguinal AT from control and *Atg5* ^eoΔ^ mice. **(F)** H&E. Staining with H&E revealed enlarged adipocytes in the inguinal, visceral, and brown AT of *Atg5* ^eoΔ^ in comparison with control mice. Scale bars, 50 μm. **(G, H)** Glucose **(G)** and insulin **(H)** tolerance were performed on control and *Atg5* ^eoΔ^ mice after a 6-hour fasting period preceding the injections of glucose or insulin (n = 3). **(I)** Glucose uptake. Insulin-stimulated 2-DGP accumulation was assessed in tissues from both control and *Atg5* ^eoΔ^. Following a 6-hour fasting period, mice received insulin injections at 0 minutes, followed by 2-DG injections at 10 minutes, and were subsequently euthanized at 30 minutes. (n = 3). **(J)** Quantitative PCR. Expression pattern of thermogenic genes evaluated in inguinal AT of control and *Atg5* ^eoΔ^. Expression levels were normalized using *Actb* and *Nono* as reference genes and compared to control mice. (n = 3). Values are means ± SEM. * *p* < 0.05; ** *p* < 0.01; *** *p* < 0.001.

The absence of *Atg5* in eosinophils resulted in an increase in body weight among female mice when compared to control mice at various time points, a finding that was observed under both normal or high-fat diets (**Figure 1A**). Notably, the two groups had no significant difference in food intake (**Figure 1B**). This is substantiated by the observed increase in AT mass and the ratio of inguinal AT to total body weight in male *Atg5* ^eoΔ^ mice (**Figures 1C, D**). Upon visual inspection of AT depots, the tissue from *Atg5* ^eoΔ^ exhibited a paler appearance and larger size, indicating notable differences compared to the control (**Figure 1E**, *inguinal AT*, and **Supplementary Figure 1A**, *three types of ATs*). Moreover, histological examination of the *Atg5* ^eoΔ^ mice using H&E staining demonstrated alterations in the cellular architecture of AT, with enlargement of the adipocytes and increased lipid droplets compared to the control (**Figure 1F**).

These morphological alterations were accompanied by impaired glucose tolerance (**Figure 1G**) and insulin tolerance (**Figure 1H**) in *Atg5* ^eoΔ^ mice compared to the control group. Insulin resistance can be discerned by the diminished glucose uptake rates in the principal insulin-responsive tissues, namely skeletal muscle, liver, and AT (38). Upon closer examination of glucose uptake rates within key insulin-sensitive tissues, we detected that the white ATs played a pivotal role in the manifestation of impaired glucose tolerance observed in *Atg5* ^eoΔ^ mice (**Figure 1I**). Considering the potential impact of eosinophils on the beiging process and observed white ATs modifications, we evaluated the expression pattern of thermogenic genes and markers of beige adipocytes. We observed downregulation of numerous thermogenic genes, prominently *Ucp1*, *Cox8b*, *Elovl3*, and marker of beige adipocytes *Cited1* and *Pat2* in inguinal AT of *Atg5* ^eoΔ^ in comparison to control mice (**Figure 1J**). The cumulative findings suggest that *Atg5* ^eoΔ^ mice exhibit an increased fat mass and impaired glucose metabolism, which can be attributed to a reduction in beiging, potentially stemming from the downregulation of thermogenic genes.

### 3.2 Increased eosinophil infiltration in adipose tissues in *Atg5* ^eoΔ^ mice

**Figure 2:**
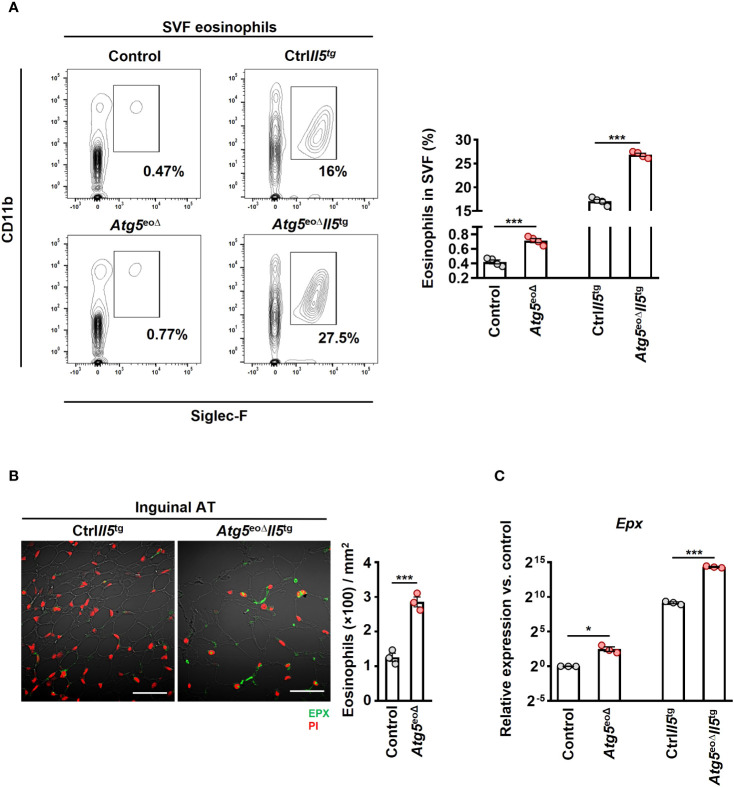
Depleting *Atg5* in eosinophils elevates eosinophil infiltration in inguinal adipose tissue. **(A)** Flow cytometry. SVF isolated from control, *Atg5* ^eoΔ^, Ctrl*Il5* ^tg^, and *Atg5* ^eoΔ^ *Il5* ^tg^ mice, and the relative numbers of eosinophils (CD11b^+^/Siglec-F^+^) were determined by flow cytometry (n = 4). **(B)** Confocal microscopy. FFPE inguinal AT sections of Ctrl*Il5* ^tg^ and *Atg5* ^eoΔ^ *Il5* ^tg^ mice were stained with monoclonal mouse anti-EPX antibody (green), and PI (red) was used for nuclear DNA visualization. (left) Representative confocal microscopy images are shown. Scale bars, 50 μm. (right) Quantification of EPX^+^ cells was performed by counting cells manually in 10 randomly selected HPF, each covering the area of 22.5×10^-3^ mm^2^, using an automatic digital slide scanner Panoramic MIDI II (n=3). **(C)** Quantitative PCR. *Epx* expression analysis of the inguinal AT of control, *Atg5* ^eoΔ^, Ctrl*Il5* ^tg^, and *Atg5* ^eoΔ^ *Il5* ^tg^ mice. Expression levels were normalized using *Actb* and *Nono* as reference genes and compared to control mice. (n = 3). Values are means ± SEM. * *p* < 0.05; *** *p* < 0.001.

Previous studies have indicated a negative correlation between obesity and the abundance of eosinophils in AT (21, 39, 40). Moreover, heightened eosinophil levels in IL-5 overexpressing mice have been linked to resistance against diet-induced obesity, elucidating a potential protective mechanism (21). In order to assess whether weight gain is driven by a decrease in eosinophil abundance in AT, we initially examined the eosinophil numbers in inguinal AT of control and *Atg5* ^eoΔ^ mice. The SVF cells were collected and subjected to flow cytometry analysis. AT-resident eosinophils were 1.7 and 1.6-fold more prevalent in *Atg5* ^eo^ ***^Δ^*** and *Atg5* ^eo^ **^Δ^** *Il5* ^tg^ inguinal AT compared to control and ctrl*Il5* ^tg^, respectively (**Figure 2A**). Given the low eosinophil count in mice adipose tissue, we employed hypereosinophilic mice for enhanced detection of eosinophils through IF staining in our experiment. Consequently, we then stained the inguinal AT of *Atg5* ^eo^ ***^Δ^*** *Il5* ^tg^ and ctrl*Il5* ^tg^ mice with an anti-EPX antibody and observed a similar increase in eosinophil infiltration in the tissue of *Atg5* ^eo^ **^Δ^** *Il5* ^tg^ mice (**Figure 2B**), supporting the data obtained from flow cytometry (**Figure 2A**). In addition, the expression of *Epx* mRNA was increased in the inguinal AT of *Atg5* ^eo^ **^Δ^** and *Atg5* ^eo^ **^Δ^** *Il5* ^tg^ when compared to the control and ctrl*Il5* ^tg^ mice, respectively (**Figure 2C**). No significant differences were observed upon evaluation of SSC, CCR3, and CD63 expression levels in adipose tissue eosinophils, which typically serve as indicators of maturity and activation levels (**Supplementary Figures 1B, C**). Taken together, eosinophil levels in AT were increased in mice with autophagy-deficient eosinophils. Therefore, it appears that the increased adiposity of these mice was the consequence of reduced functional activity of eosinophils within AT and not owing to reduced eosinophil numbers.

### 3.3 The absence of *Atg5* is associated with arginase type II expression in eosinophils, resulting in reduced beiging in adipocytes

**Figure 3:**
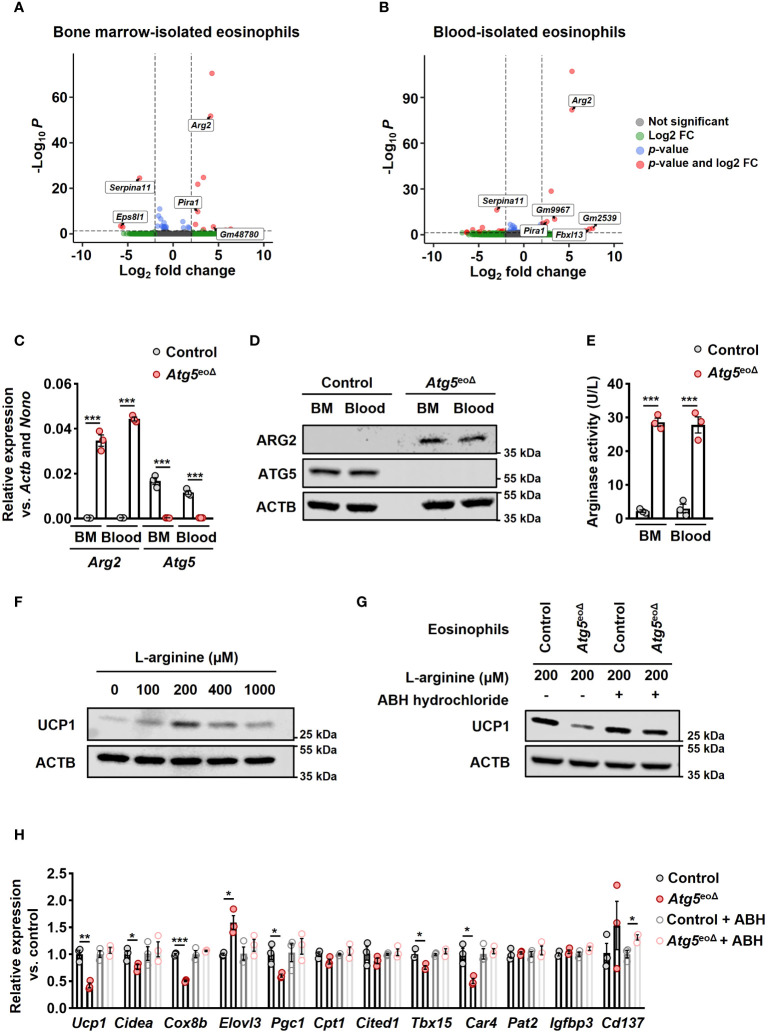
ATG5 deficiency in eosinophils induces *Arg2* expression, resulting in decreased beiging in adipocytes. **(A, B)** RNA-seq. The volcano plot of differentially expressed genes (DEGs) in bone marrow **(A)** and blood **(B)**-isolated eosinophils from *Atg5* ^eoΔ^ *Il5* ^tg^ was compared to bone marrow and blood-isolated eosinophils from Ctrl*Il5* ^tg^. A total of 13 and 25 DEGs were identified, respectively. **(C)** Quantitative PCR. The isolation of eosinophils from bone marrow (BM) and blood of Ctrl*II5* ^tg^ and *Atg5* ^eoΔ^ *II5* ^tg^ mice was followed by gene expression analysis for *Arg2* and *Atg5*. The expression of the target genes was normalized using *Actb* and *Nono* as reference genes (n=3). **(D)** Immunoblotting. Protein lysates from freshly isolated BM and blood-derived eosinophils of Ctrl*II5* ^tg^ and *Atg5* ^eoΔ^ *II5* ^tg^ mice were subjected to analysis for ATG5 and ARG2 protein expression. ACTB protein levels were utilized as loading controls. **(E)** Arginase activity assay. Eosinophils freshly isolated from BM and blood samples of Ctrl*II5* ^tg^ and *Atg5* ^eoΔ^ *II5* ^tg^ mice were subjected to an arginase activity assay (n = 3). **(F)** Immunoblotting. Protein lysates were obtained from mature adipocytes incubated in L-arginine free DMEM supplemented with different L-arginine concentrations for five hours. ACTB protein levels served as loading controls. **(G)** Immunoblotting. Eosinophils were cultured in the presence and absence of ABH-hydrochloride, and their supernatants were applied to adipocytes, followed by UCP1 immunoblotting. **(H)** Quantitative PCR. qPCR was performed on adipocytes that underwent the same treatments described in the previous section to evaluate the expression level of thermogenic genes. Expression levels were normalized using *Actb* and *Nono* as reference genes and compared to control mice. (n = 3). Values are means ± SEM. * *p* < 0.05; ** *p* < 0.01; *** *p* < 0.001.

To better understand the molecular basis underlying the role of autophagy in eosinophils, we performed total RNA-seq in control and *Atg5*-knockout eosinophils isolated from bone marrow and blood of *Atg5* ^eo^ **^Δ^** *Il5* ^tg^ and ctrl*Il5* ^tg^ mice. The purity of the isolated eosinophils is depicted in **Supplementary Figure 2C**. Using DESeq2 for differential expression analysis, we identified 10 up-regulated and 3 down-regulated genes in bone marrow-isolated eosinophils of *Atg5* ^eo^ **^Δ^** *Il5* ^tg^ compared to ctrl*Il5* ^tg^. In blood-isolated eosinophils of *Atg5* ^eo^ **^Δ^** *Il5* ^tg^ compared to ctrl*Il5* ^tg^, we found 12 up-regulated and 13 down-regulated genes (adjusted *p* value < 0.05; |log2 fold change| > 2) (**Figures 3A, B**, and **Supplementary Figures 2A, B**) (GEO accession GSE245134). Of note, arginase type II (*Arg2*) was one of the most up-regulated genes in both bone marrow and blood eosinophils (log2FC = 4.1 and 5.3, respectively). It has been shown that an increase in *Arg2*, which hydrolyzes L-arginine to urea and L-ornithine, is involved in cardiovascular disease conditions by reducing L-arginine availability. Inversely, the deletion of *Arg2* leads to an upregulation of *Ucp1* (41). We further validated the expression of *Arg2* mRNA by qPCR in eosinophils isolated from the blood and bone marrow of *Atg5* ^eo^ **^Δ^** *Il5* ^tg^ and ctrl*Il5* ^tg^ mice (**Figure 3C**). Moreover, the immunoblot analysis confirmed the upregulation of ARG2 also at the protein level (**Figure 3D**). Intriguingly, the arginase activity was detected exclusively in *Atg5*-knockout eosinophils and was absent in control eosinophils (**Figure 3E**).

Arginase 2 can impact L-arginine levels by catalyzing the conversion of L-arginine into L-ornithine and urea (42). L-arginine has been shown to promote the development of brown AT in fetal lambs from obese ewes (43). Furthermore, brown adipocyte precursor cells exhibit concentration-dependent responses to L-arginine, affecting their growth, protein turnover, and cell signaling pathways. An optimal extracellular concentration of 200 μmol for UCP1 expression, equivalent to L-arginine concentrations in the maternal plasma of obese ewes receiving L-arginine supplementation, has been reported (44). We conducted experiments to assess the impact of varying L-arginine concentrations on murine mature adipocytes. Our findings also indicate a concentration-dependent relationship between the L-arginine concentration and the expression of thermogenic genes, with an optimal extracellular concentration of 200 μmol (**Figure 2F** and **Supplementary Figure 2D**). To assess the impact of *Atg5* in eosinophils on mature adipocytes, along with exploring the role of *Arg2*, we cultured control and *Atg5*-knockout eosinophils in L-arginine free DMEM supplemented with 200 μmol L-arginine in the presence and absence of the arginase inhibitor ABH-hydrochloride, and subsequently, adipocytes were treated with eosinophils’ supernatants (**Supplementary Figure 3A**). The results from immunoblot analysis for expression of UCP1 (**Figure 3G**) and mRNA expression analyses of thermogenic genes (**Figure 3H**) clearly indicate that *Atg5*-knockout eosinophils significantly reduce adipocyte beiging, and the effect can be attenuated by suppressing arginase activity. These findings suggest that defective autophagy in eosinophils is associated with an upregulation of *Arg2*, which suppresses the process of adipocyte beiging.

### 3.4 The absence of *Atg5* in eosinophils is associated with an induction of IFNγ expression in macrophages, resulting in reduced beiging in adipocytes

**Figure 4:**
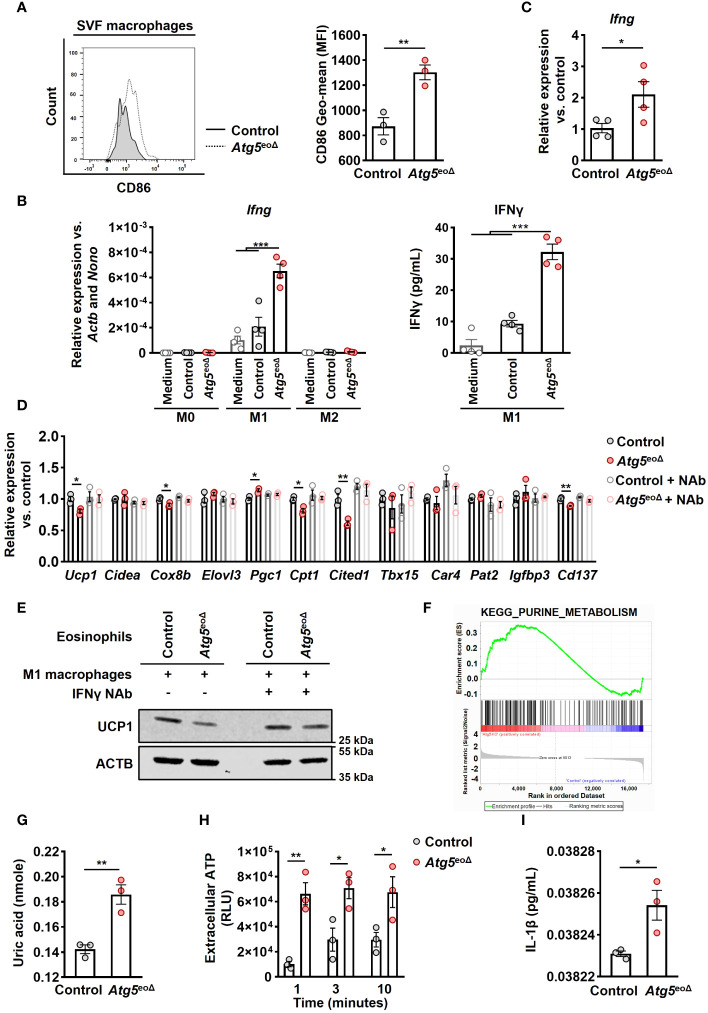
*Atg5*-knockout eosinophils trigger IFNγ expression in macrophages, resulting in the inhibition of beiging. **(A)** Flow cytometry. Representative original flow cytometry data of CD86 expression in macrophages (CD11b^+^ and F4/80^+^) within the SVF of control and *Atg5* ^eoΔ^ mice are presented. (Right) CD86 expression levels (Geometric mean fluorescence intensity) were quantified in macrophages (n=3). **(B)** (left) Quantitative PCR. *Ifng* expression levels were measured by qPCR on macrophages polarized in medium alone or in the presence of either control or *Atg5*-knockout eosinophils. Expression levels were normalized using *Actb* and *Nono* as reference genes and compared to the medium alone conditions (n=4). (right) ELISA measurements. IFNγ concentrations were measured in the supernatant of macrophages polarized under previous conditions (n=4). **(C)** Quantitative PCR. *Ifng* expression levels in inguinal AT were measured by qPCR, normalized using *Actb* and *Nono* as reference genes, and compared to the control mice (n=4). **(D)** Quantitative PCR. Macrophages were polarized in the presence of either control or *Atg5*-knockout eosinophils for 24 hours, with or without the presence of an IFNγ neutralization antibody (NAb). Subsequently, adipocytes were treated with the resulting supernatant for five hours, followed by qPCR analysis of thermogenic genes. The expression levels were normalized with *Actb* and *Nono* and compared to the control condition (n = 3). **(E)** Immunoblotting. UCP1 expression in adipocytes subjected to the same treatments described in the previous section was evaluated. **(F)** Pathway analysis. Purine metabolism pathway were among the enriched pathways. Enrichment analysis was performed with the KEGG curated gene set collection by GSEA. **(G)** Uric acid. Freshly isolated eosinophils from both Ctrl*II5* ^tg^ and *Atg5* ^eoΔ^ *II5* ^tg^ mice underwent quantification of uric acid levels (n = 3). **(H)** ATP release. ATP in cell-free supernatant of control and *Atg5*-knockout eosinophils was measured by ATP-dependent luciferase activity using an ATP determination kit (n = 3). **(I)** ELISA measurements. IL-1β concentrations were measured in the supernatant of macrophages polarized in the presence of either control or *Atg5*-knockout eosinophils (n=3). Values are means ± SEM. * *p* < 0.05; ** *p* < 0.01; *** *p* < 0.001.

Previous reports have shown the significance of eosinophils in AT for sustaining alternatively activated AT-macrophages and regulating inflammation in white AT associated with obesity-related metabolic disorders (21, 45, 46). Therefore, we investigated the influence of *Atg5*-knockout eosinophils on macrophages. While we did not observe any alterations in the abundance of inguinal AT-macrophages (**Supplementary Figure 4A**), we detected an increase in CD86 levels, a marker of M1 macrophages (47) (**Figure 4A**). To evaluate the influence of *Atg5*-knockout eosinophils on macrophage cytokine signaling, we examined the expression of various pro-inflammatory and anti-inflammatory genes in macrophages co-cultured with either control or *Atg5*-knockout eosinophils. mRNA expression analysis showed an upregulation of *Ifng* in macrophages co-cultured with *Atg5*-knockout eosinophils compared to those co-cultured with control eosinophils or medium (**Figure 4B** *left panel* and **Supplementary Figure 4B**). This observation was confirmed by quantifying interferon-γ (IFNγ) protein concentrations in the co-cultured supernatants using ELISA assay (**Figure 4B**, *right panel*). Additionally, we examined whether a similar increase in mRNA expression of *Ifng* occurred in the inguinal AT of *Atg5* ^eoΔ^ compared to control mice. Indeed, we also observed an upregulation of *Ifng* mRNA in inguinal AT of *Atg5* ^eoΔ^ mice, supporting our findings under *in vivo* conditions (**Figure 4C**).

To investigate the functional role of IFNγ, we induced macrophage polarization in the presence and absence of either control eosinophils or eosinophils lacking *Atg5* for 24 hours. This co-culturing process was carried out in the presence and absence of an IFNγ NAb, and adipocytes were then exposed to the resulting supernatants (**Supplementary Figure 3B**). As depicted in **Figure 4D**, macrophages stimulated with the conditioned media from *Atg5*-knockout eosinophils reduced beiging in adipocytes. This effect was not observed in the presence of the IFNγ NAb (**Figure 4D**). Immunoblot analysis of UCP1 protein expression in adipocytes confirmed the mRNA expression results (**Figure 4E**).

To understand the mechanism by which autophagy deficiency in eosinophils induces IFNγ production in macrophages, we utilized Gene Set Enrichment Analysis (GSEA) on gene expression data from both control and *Atg5*-knockout eosinophils after normalization. The data analysis revealed a notable enrichment of purine metabolism in *Atg5*-knockout eosinophils (**Figure 4F**). Subsequently, we examined the concentration of uric acid, which is the end product of the purine metabolic pathway (48). A significant increase in uric acid levels was observed in *Atg5*-knockout compared to control eosinophils (**Figure 4G**). Uric acid has been documented as an endogenous danger signal for eosinophils, prompting the release of ATP (49). Thus, we investigated the release of ATP from both control and *Atg5*-knockout eosinophils and observed an elevated ATP release at various time points in *Atg5*-knockout eosinophils (**Figure 4H**). Both uric acid and extracellular ATP can activate the NLRP3 inflammasome, increasing IL-1β production (50, 51). Consistent with this, we observed a significant rise in IL-1β levels in the supernatant of macrophages polarized in the presence of *Atg5*-knockout eosinophils compared to those polarized with control eosinophils (**Figure 4I**).

In summary, these findings suggest that defective autophagy in eosinophils results in increased uric acid and extracellular ATP release, which in turn induce inflammatory cytokines, such as IL-1β and IFNγ. These factors collectively contribute to a phenotypic shift in macrophages toward the inflammatory M1 subtype, which inhibits adipocyte beiging.

## 4 Discussion

Obesity is characterized by an imbalance between white and brown adipose tissues. Obesity significantly elevates the risk of all-cause mortality, including cardiovascular diseases and cancers. Notably, brown and beige ATs are potential therapeutic targets in obese people owing to their inherent thermogenic capabilities and potential to enhance glucose metabolism (52, 53). Immune cells are known to regulate the thermogenic activity of brown and beige ATs (21, 54). While macrophages have received ample attention, a recent study challenged their previously defined role in adaptive thermogenesis via catecholamine production (55). More recently, several studies have obtained evidence for a role of eosinophils in maintaining AT homeostasis (56, 57).

We have previously shown that autophagy positively influences the proliferation of eosinophil precursors but reduces the levels of degranulation and bacterial killing in mature eosinophils (16). In this study, utilizing mice with eosinophil-specific *Atg5* gene deficiency, we observed elevated body weight, impaired glucose metabolism, and reduced beiging. Furthermore, glucose uptake assays conducted on inguinal AT, visceral AT, brown AT, liver, and muscle tissues revealed that white ATs (inguinal AT and visceral AT) exhibited impaired glucose uptake in *Atg5* ^eo^ **^Δ^** compared to control mice. These findings suggest a crucial role of autophagy in eosinophils for maintaining WAT homeostasis and the regulation of thermogenesis. To understand how autophagy in eosinophils influences the adipose tissue metabolism in *Atg5* ^eoΔ^ mice, we examined AT-eosinophil levels, eosinophils-adipocytes interactions, and their effects on AT-macrophages.

Eosinophil levels have been reported to correlate negatively with body weight, resulting in fewer eosinophils within the AT of obese individuals (58). The exposure to cold temperatures, a condition known to stimulate beiging, increases the proportion of eosinophils in AT (57). Despite the delayed maturation in the bone marrow and reduced eosinophil counts observed in blood and various tissues of *Atg5* ^eoΔ^ mice (16), our observations revealed elevated eosinophil counts exhibiting similar maturation and degranulation activity levels in *Atg5* ^eoΔ^ compared to control mice while displaying reduced beige AT in this model. This discrepancy suggests a potential involvement of a compensatory mechanism or altered trafficking dynamics specific to adipose tissue in response to *Atg5* deficiency, prompting further investigation into the intricate regulatory pathways governing eosinophil distribution and function in this unique context. Moreover, our report points to a significant role of autophagy in modulating the functional activity of eosinophils within AT. These observations are in agreement with a recent study, suggesting that the beneficial role of eosinophils in beiging may be more dependent on their functions rather than their numbers, as artificially increasing eosinophil levels in AT using recombinant IL-5 did not result in metabolic benefits (23).

RNA-seq analysis revealed upregulation of *Arg2* in *Atg5*-knockout eosinophils, which was further confirmed by mRNA expression analysis, immunoblotting, and arginase activity assays. It has been shown that the upregulation of arginase, a urea hydrolase enzyme with two isoforms (*Arg1*-cytosolic and *Arg2*-mitochondrial), results in a decrease in L-arginine bioavailability, which is implicated in pathologies associated with obesity and diabetes (59, 60). We observed a concentration-dependent correlation between L-arginine levels and the expression of thermogenic genes in mature murine adipocytes, with the most favorable extracellular concentration being 200 μmol. This finding is in agreement with a prior report on brown adipocyte precursor cells isolated from fetal lambs (44). Interestingly, we observed that conditioned media from *Atg5*-knockout eosinophils reduced adipocyte beiging compared to those from control eosinophils. This effect was blunted by inhibiting arginase activity using a specific inhibitor. Therefore, increased arginase activity provided by autophagy-deficient eosinophils results in increased body weight.

We also found that autophagy deficiency in eosinophils induces a shift in macrophage behavior towards a pro-inflammatory state indicated by increased IFNγ expression. This alteration in macrophages results in reduced beiging of adipocytes. Our observations are in agreement with prior studies that have demonstrated brown/beige AT inflammation is associated with obesity and metabolic diseases (61). Mechanistically, autophagy deficiency in eosinophils leads to the enrichment of purine metabolism, resulting in elevated uric acid levels and increased ATP release. As a result, uric acid and extracellular ATP could activate the NLRP3 inflammasome and augment local IL-1β production, which acts as a co-stimulator of interferon-γ production (62). These findings emphasize the intricate interplay between the immune system and AT in metabolic regulation, providing insights into potential mechanisms underlying obesity-related metabolic disorders.

**Figure 5:**
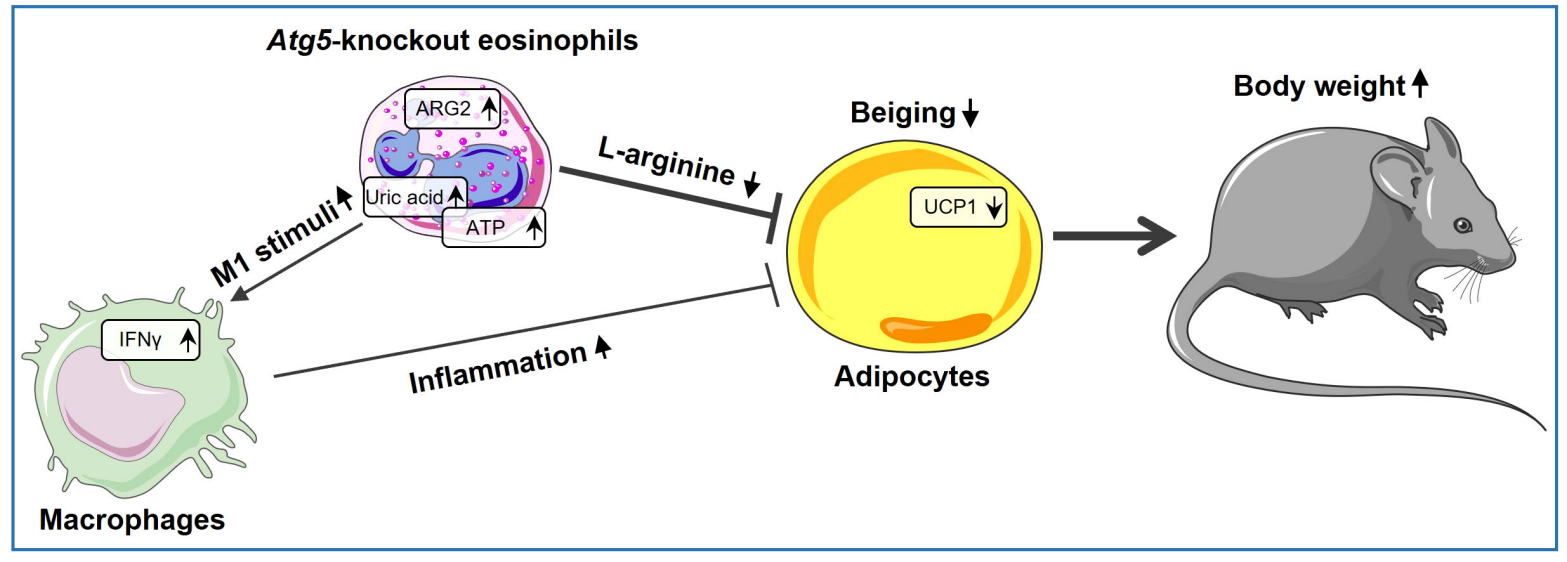
*Atg5*’s role in eosinophils shaping beige adipocyte development. ARG2 is upregulated in *Atg5*-knockout eosinophils, leading to decreased L-arginine bioavailability and reduced adipocyte beiging. Simultaneously, eosinophil autophagy deficiency enriches purine metabolism, resulting in elevated uric acid levels and increased ATP release. As a result, uric acid and extracellular ATP can activate the NLRP3 inflammasome, augmenting local IL-1β production and increasing IFNγ expression, thereby inducing a shift in macrophage behavior toward a pro-inflammatory state, which decreases adipocyte beiging.

Taken together, our study provides direct evidence for the involvement of ATG5 in eosinophils in inducing beiging and reducing adiposity, both by directly reducing L-arginine levels due to increased arginase activity and indirectly by creating a heightened inflammatory environment, as illustrated in our proposed model (**Figure 5**). In this scenario, the direct effect of eosinophils on adipocytes through the expression of *Arg2* might be more important than the indirect effect through macrophages, as assessed by expression level of thermogenic genes. Our findings demonstrate that autophagy regulates the functional activity of eosinophils to maintain adipose tissue homeostasis. However, it is worth noting that ATG5 can also play a role in functions that are not reliant on autophagy (63). Therefore, although we have previously shown that *Atg5*-knockout eosinophils lack autophagy (16), but some of the observed effects in this report might be regulated by ATG5 in an autophagy-independent manner. Nevertheless, the findings of this study point to a direct role of eosinophils in regulating adipocytes, besides regulating macrophages, and add new information, which may provide potential new avenues for innovative therapeutic approaches.

## Data availability statement

The raw sequence data have been deposited in the Gene Expression Omnibus (GEO), accession number GSE245134. https://www.ncbi.nlm.nih.gov/geo/query/acc.cgi?acc=GSE245134.

## Ethics statement

The animal study was approved by Veterinary Office of the Canton of Bern and conducted under Swiss federal legislation on animal welfare under animal license number BE26/2021. The study was conducted in accordance with the local legislation and institutional requirements.

## Author contributions

AH: Formal analysis, Investigation, Methodology, Software, Validation, Visualization, Writing – original draft. NG: Investigation, Methodology, Writing – original draft. NM: Investigation, Methodology, Writing – original draft. DS: Methodology, Writing – original draft. KO: Methodology, Writing – original draft. SY: Conceptualization, Formal analysis, Funding acquisition, Investigation, Methodology, Supervision, Writing – review & editing. HS: Conceptualization, Funding acquisition, Investigation, Project administration, Resources, Supervision, Writing – review & editing.
