# Supplementary material for: The regulatory role of eosinophils in adipose tissue depends on autophagy
Source: Front Immunol. 2024 Jan 3;14:1331151. doi: 10.3389/fimmu.2023.1331151 (PMC10792036; doi:10.3389/fimmu.2023.1331151)
Supplement: Supplementary file 1 [file DataSheet_1.docx]

Supplementary Material

The Regulatory Role of Eosinophils in Adipose Tissue Depends on Autophagy

Aref Hosseini^1^, Nina Germic^1^, Nikita Markov^1^, Darko Stojkov^1^, Kevin Oberson^1^, Shida Yousefi^1^, Hans-Uwe Simon^1,2*^

^1^ Institute of Pharmacology, University of Bern, Bern, Switzerland.

^2^ Institute of Biochemistry, Brandenburg Medical School, Neuruppin, Germany.

*** Correspondence:**Hans-Uwe Simon

**Supplementary Figure count: 4**


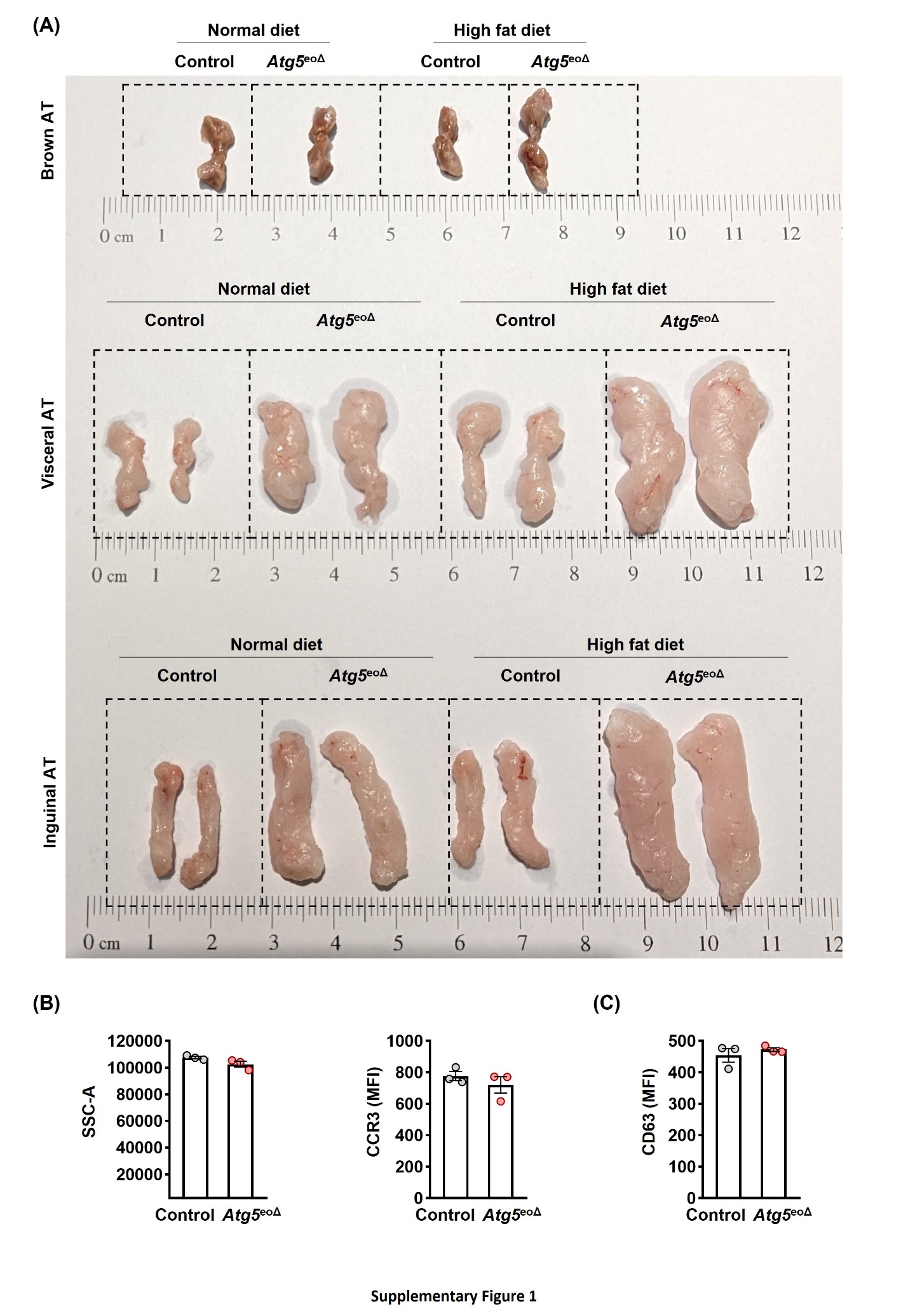


**Supplementary Figure 1. Visual representation of brown, visceral, and inguinal ATs in control and *Atg5*^eoΔ^ mice and phenotype of eosinophils in adipose tissue.**

(A) Comparisons of brown, visceral, and inguinal ATs between control and *Atg5*^eoΔ^ mice under either normal or high-fat diet. (B) Flow cytometry. Eosinophils in SVF were analyzed for their maturity and granularity status using CCR3 and SSC-A, respectively (n = 3). (C) Flow cytometry. The activity of eosinophils in SVF was analyzed by utilizing CD63 expression as a degranulation marker (n = 3).


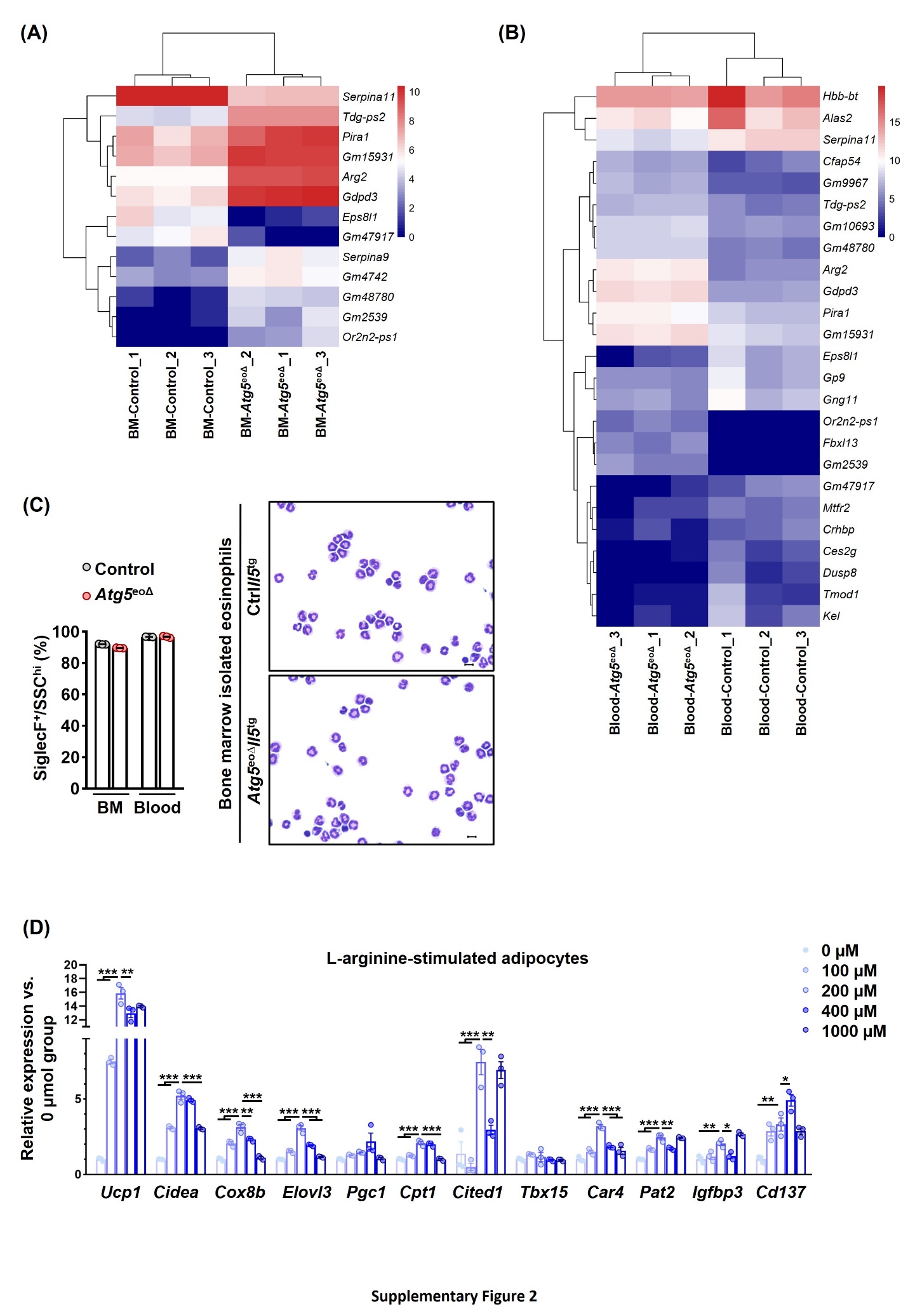


**Supplementary Figure 2. *Arg2* is upregulated in *Atg5*-knockout eosinophils, and L-arginine concentration affects beiging in adipocytes.**

(A and B) RNA-seq. To compare DEGs, the heatmaps for eosinophils isolated from bone marrow (A) and blood (B) in *Atg5*^eo∆^*Il5*^tg^ were contrasted with those from Ctrl*Il5*^tg^. (C) Flow cytometry and cytospin. (left) Purity of eosinophils (SSC^hi^/SiglecF^+^) isolated from bone marrow and blood of CtrlIl5^tg^, and Atg5^eo∆^Il5^tg^ mice for RNA-seq analysis (n = 3). (right) Representative cytospin images are shown. Scale bars, 10 μm. (D) Quantitative PCR. qPCR was conducted on mature adipocytes incubated in L-arginine free DMEM supplemented with various L-arginine concentrations for five hours. Expression levels were normalized using *Actb*and *Nono*as reference genes and compared to 0 μmol L-arginine group (n = 3). Values are means ± SEM. * *p* < 0.05; ** *p* < 0.01; *** *p* < 0.001.


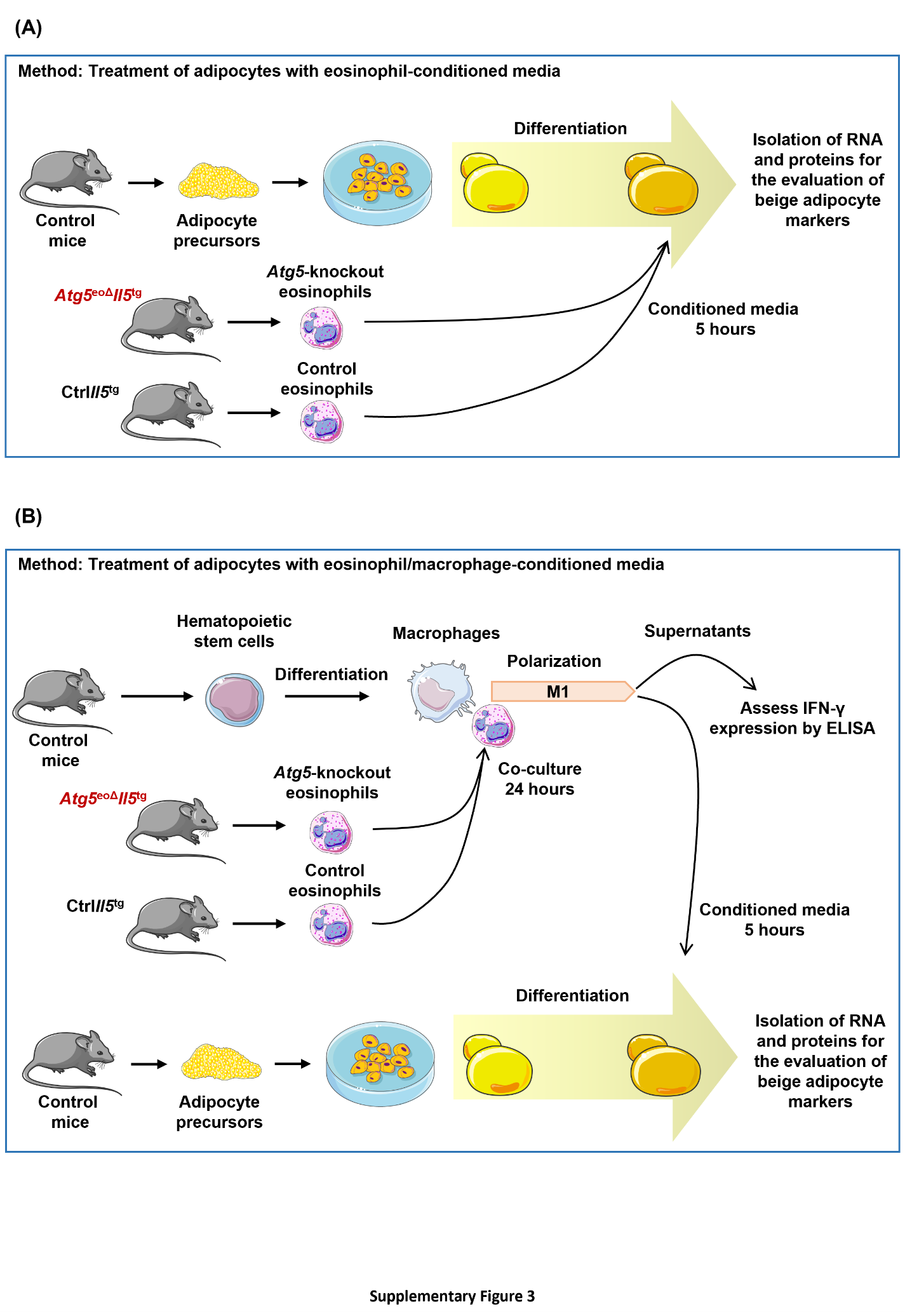


**Supplementary Figure 3. Flow scheme of adipocytes treatment with conditioned media.**

(A) Adipocyte treatment with eosinophil-conditioned media. Adipocyte precursors were obtained from inguinal AT harvested from control mice. Primary murine preadipocytes were differentiated for six days and then treated with conditioned media for 5 hours. Eosinophils were isolated from the bone marrow of Ctrl*II5*^tg^ and *Atg5*^eoΔ^*II5*^tg^ mice. The conditioned media was prepared by incubating eosinophils in L-arginine free DMEM supplemented with 200 μmol L-arginine for 5 hours. Subsequently, the supernatant was collected and centrifuged to remove cells. After 5 hours of incubation of adipocytes with the conditioned media, cells were collected for RNA and protein extraction. (B) Adipocyte treatment with eosinophils-macrophages-conditioned media. Adipocytes and eosinophils were prepared as described in the previous section. Additionally, mouse bone marrow cells were harvested and differentiated into macrophages over eight days. Following this, the macrophages were polarized toward M1 macrophages in the presence of either control or *Atg5*-knockout eosinophils in DMEM/F12 media for 24 hours. As in the preceding section, adipocyte precursors were treated with the conditioned media on day six for 5 hours. Finally, the adipocytes were collected for RNA and protein extraction.


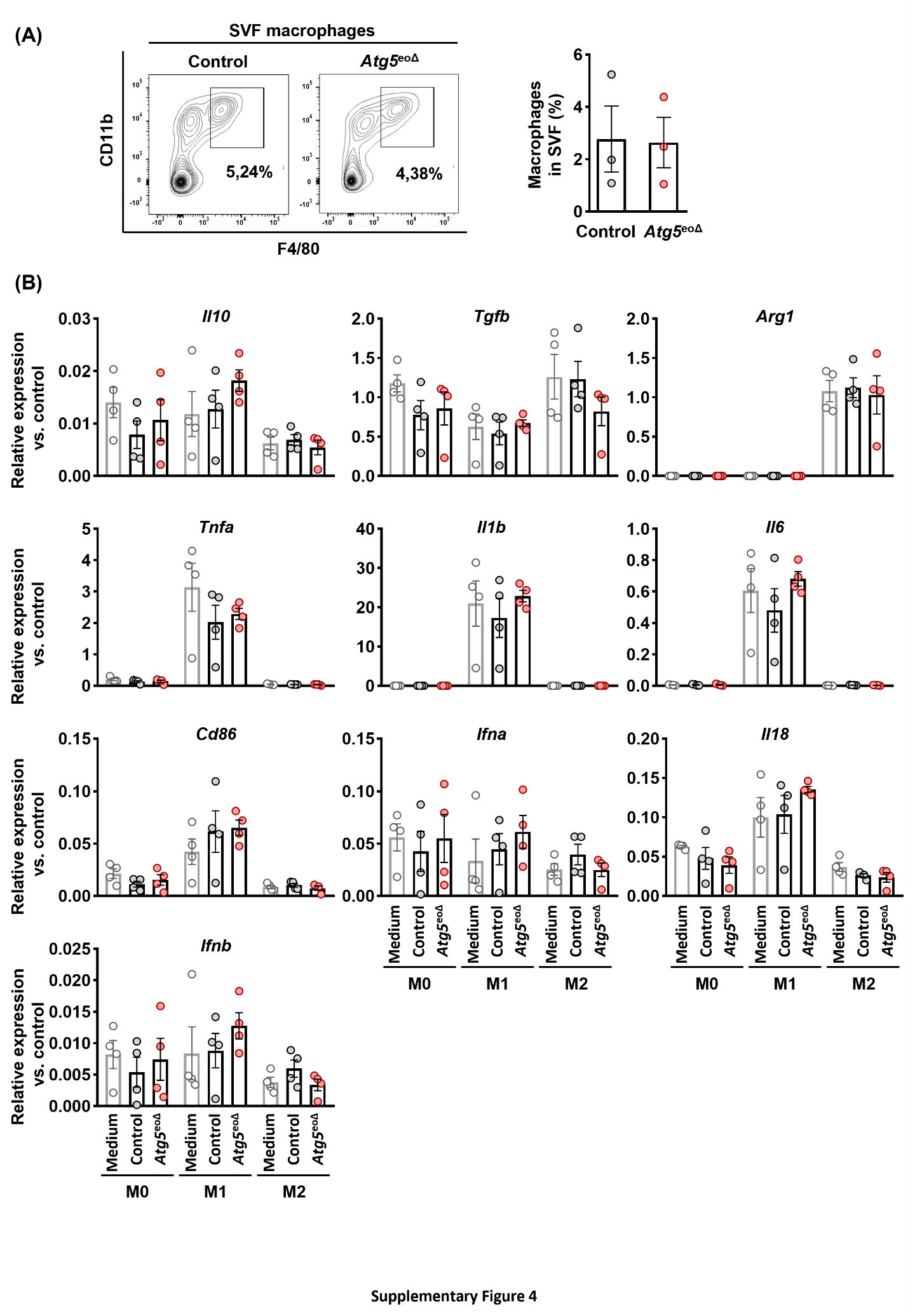


**Supplementary Figure 4. Concentration of macrophages in SVF; and impact of control and *Atg5*-knockout eosinophils on macrophage cytokine signaling.**

(A) Flow cytometry. SVF was isolated from control and *Atg5*^eo∆^, and the relative numbers of macrophages (CD11b^+^/F4/80^+^) were determined by flow cytometry (n = 3). (B) Quantitative PCR. qPCR was used to measure the expression levels of pro-inflammatory and anti-inflammatory genes in macrophages. These macrophages were polarized in the medium alone or the presence of either control or *Atg5*-knockout eosinophils. Expression levels were normalized using *Actb* and *Nono* as reference genes and compared to the medium conditions (n = 4).
